# Short-term outcomes of periacetabular osteotomy versus periacetabular osteotomy with concomitant femoral osteochondroplasty: a propensity matched analysis

**DOI:** 10.1093/jhps/hnae046

**Published:** 2024-12-18

**Authors:** Nathan V Houlihan, Daniel J Sucato, Tanner Thornton, Jeffrey J Nepple, John C Clohisy, Wudbhav N Sankar

**Affiliations:** Division of Orthoapedic Surgery, The Children’s Hospital of Philadelphia, 34th and Civic Center Blvd, Philadelphia, PA 19104, United States; Texas Scottish Rite Hospital for Children, 2222 Welborn St, Dallas, TX 75219, United States; Department of Orthopaedic Surgery, Washington University, 1044 N. Mason Rd, Suite 110, St Louis, MO 63141, United States; Department of Orthopaedic Surgery, Washington University, 1044 N. Mason Rd, Suite 110, St Louis, MO 63141, United States; Department of Orthopaedic Surgery, Washington University, 1044 N. Mason Rd, Suite 110, St Louis, MO 63141, United States; Division of Orthoapedic Surgery, The Children’s Hospital of Philadelphia, 34th and Civic Center Blvd, Philadelphia, PA 19104, United States

## Abstract

This study compared outcomes of periacetabular osteotomy (PAO) with and without femoral osteochondroplasty (OCP) in treating symptomatic acetabular dysplasia through propensity score matching. Data from a prospective multicenter cohort of patients undergoing PAO from 2007 to 2014 were analyzed. Inclusion criteria were a lateral center edge angle <25°. The exclusion criteria were history of previous procedure and age >45 years. A 2- to 5-year follow-up interval was utilized; patients outside this follow-up window were excluded. Propensity matching variables included sex, baseline hip internal rotation at 90° flexion, preoperative alpha angle, lateral center edge angle, modified Harris Hip score (mHHS), and arthroscopy at the time of surgery. Propensity scores were calculated using logistic regression with treatment as the dependent variable. Clinical failure was defined as failure to meet the minimal clinically important difference and patient acceptable symptom state for mHHS or a need for reoperation. There were 219 patients that met the inclusion criteria. Of these, 116 patients were matched, representing 58 pairs (PAO/OCP = 58; PAO without OCP = 58). Preoperative functional scores were similar between groups. At mean 4.1 years follow-up, there were no significant differences in the rates of clinical failure or reoperation between the two groups [PAO/OCP = 13 (22%), PAO without OCP = 8 (14%); *P* = .23] Similarly, the final mHHS was 83.2 ± 16.2 for the PAO/OCP group and 84.1 ± 15.9 for the isolated PAO group, with no significant difference (*P* = .74). In the treatment of symptomatic acetabular dysplasia, isolated PAO is noninferior to combined PAO/OCP at short-term follow-up in patients who are likely to be treated by either method.

## Introduction

Symptomatic acetabular dysplasia is commonly treated with periacetabular osteotomy (PAO) in adolescents and young adults [1, 2]. PAO is a safe and effective treatment option with favorable outcomes for even mild or borderline dysplasia [3–6]. While the clinical outcomes of PAO are well-studied, there remains controversy as to the utility of concomitant procedures to treat coexisting hip pathology [7–10]. One such pathology is femoroacetabular impingement (FAI), which may either coexist with acetabular dysplasia or develop iatrogenically subsequent to repositioning of the acetabulum [11]. The reported rate of symptomatic FAI following PAO varies widely (12%–48%) and includes both pre-existing FAI, typically caused by CAM lesions, and iatrogenic FAI, which may occur due to acetabular retroversion or overcorrection during PAO [12, 13]. Symptomatic FAI following PAO has been associated with the need for reoperation [14]. As a result, some surgeons elect to perform osteochondroplasty (OCPs) at the time of PAO to mitigate anticipated postoperative FAI. Indications for these osteoplasties are unclear, but surgeons often base their decisions on internal rotation range of motion after PAO following acetabular correction or based on underlying proximal femoral morphology [15].

Previous studies comparing the outcomes of PAO with concomitant OCP and PAO without OCP have yielded conflicting results [9, 16, 17]. Wells *et al*., in a long-term study of 154 hips followed for a mean of 10.3 years, demonstrated that the combined PAO/OCP procedure was protective against clinical failure compared to PAO alone, with significantly better joint survival [17]. In contrast, Goronzy *et al*., in a study with a shorter follow-up of 63 ± 18 months, found no significant difference in outcomes Western Ontario and McMaster Universities Arthritis Index [WOMAC scores and conversion to total hip arthroplasty (THA)] between the two groups [9]. However, this study did show improved alpha angles in the PAO/OCP group. One of the potential pitfalls in the interpretation of these studies is the lack of preoperative matching in both, which may result in postoperative comparisons between patient groups with different baseline characteristics. Scott *et al*. further highlighted the importance of addressing CAM deformities, noting that alpha angles >60° are associated with increased joint contact stress after PAO [16]. Thus, the question remains whether preoperative baseline differences, such as CAM deformity, might explain the discrepancies in these outcomes.

The purpose of this study was to compare the functional outcomes of surgical treatment of symptomatic acetabular dysplasia with isolated PAO and PAO combined with OCP using propensity score matching.

## Materials and methods

This study utilized data from a prospective multicenter cohort of patients undergoing PAO from 2007 to 2014 with outcome scores within a 2- to 5-year follow-up window. Institutional review board approval was obtained for the study. Surgeries were performed by 11 surgeons at 9 institutions. All surgeons were experienced in PAO surgery, and the decision to perform a PAO/OCP versus PAO without OCP was determined by the treating surgeon. All PAOs were performed with the modern Bernese technique using abductor and rectus-sparing approaches. For those patients who required an open osteochondroplasty, the iliocapsularis muscle was elevated off of the anterior capsule. [2, 18] During the combined procedure, open OCP was performed after acetabular repositioning. An I-shaped arthrotomy was made on the anterior femoral neck, lateral to the rectus femoris through the PAO incision. The bone was then recessed using a combination of osteotomes and a burr under fluoroscopic guidance. Intraoperative motion was then rechecked to ensure adequate bony recession. Anatomic capsular closure was routine.

Inclusion criteria for the present study were a diagnosis of acetabular dysplasia [defined as a lateral center edge angle (LCEA) <25°], postoperative follow-up between 2 and 5 years, and the absence of any pertinent underlying syndrome (e.g. Down syndrome, cerebral palsy, Charcot-Marie-Tooth disease, etc.) or prior surgery. Exclusion criteria were a history of previous ipsilateral hip procedures and age >45 years at time of surgery. Patients who underwent hip arthroscopy at the time of PAO were not automatically excluded, as we felt that this is a commonly performed procedure to address coexisting chondrolabral pathology, and excluding this concomitant operation would compromise the generalizability of our results. All hip arthroscopies were performed for diagnostic purposes or to address chondrolabral injury; no OCPs were performed arthroscopically in this series. However, patients who underwent proximal femoral osteotomy, surgical hip dislocation, acetabular or femoral head chondroplasty, acetabular or femoral head microfracture, or ligamentum teres debridement were excluded, as these additional procedures were felt to potentially influence the outcome after PAO.

**Figure 1.**
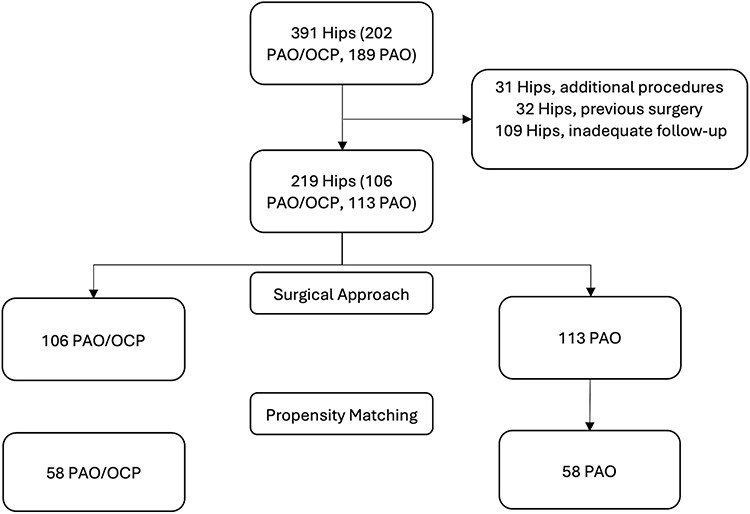
Consort diagram for propensity matching.

A total of 391 hips that underwent primary surgical treatment for acetabular dysplasia met the inclusion criteria for this study. There were 31 patients excluded due to the presence of additional surgical procedures at the time of PAO. An additional 32 patients were excluded due to a history of previous ipsilateral hip surgery, and 109 patients were excluded due to inadequate follow-up. This included patients lost to follow-up or not having reached the required 2–5 years post-surgery. After exclusions, there were 219 patients available for matching (Fig. 1). Statistical analysis was performed using STATA version 14.0, under the supervision of a trained statistician. Propensity score matching is a statistical method used to minimize bias and confounding in nonrandomized studies by ensuring similarity in baseline characteristics between treatment groups. This type of analysis allows for statistically valid comparison of outcomes in nonrandomized studies. Propensity scores (from 0.00 to 1.00) were estimated using logistic regression with treatment (PAO with OCP versus PAO without OCP) as the outcome and factors thought to play a role in disease severity, surgical decision-making, and clinical prognosis as independent variables. These independent covariates included sex, baseline internal hip rotation at 90° flexion (IRF), preoperative alpha angle (i.e. maximum alpha angle on any view), preoperative LCEA, baseline modified Harris Hip score (mHHS), and concomitant hip arthroscopy. PAO with OCP and PAO without OCP cases were matched 1:1 to minimize standardized differences of the matching variables. To assess the quality of the match results, standardized differences with a caliper of 0.1 were used to determine that there were no significant correlations between the groups and each covariate. There were 219 patients (106 who underwent PAO with OCP and 113 who underwent PAO without OCP) eligible to be matched. After the match, 116 patients were included in the study and analysis.

Outcomes were determined using patient-reported outcome measures (PROMs). The primary outcome for this study was need for reoperation or clinical failure. Reoperation was defined as revision surgery or conversion to THA. Implant removal was not considered a form of reoperation for the purposes of this study. Clinical failure was defined as failure to meet both the minimal clinically important difference (MCID) and the patient acceptable symptom state (PASS) of the mHHS at 2- to 5-year follow-up. Cases where either the MCID or PASS was met were not considered clinical failures. The MCID of mHHS is 8, and PASS is a score greater than 74. [19] Secondary outcomes were HOOS (Hip Disability and Osteoarthritis Outcome Score) subscores.

Outcomes were compared between propensity matched groups. Wilcoxon Signed-Rank tests were used to assess differences in postoperative outcomes between matched pairs. McNemar’s test was used to assess the rates of clinical failure. If one or both subjects of a matched pair were missing a secondary outcome measure, then the match did not contribute to the calculation of paired mean differences.

## Results

There were 116 hips included in the cohort representing 58 propensity-matched pairs (PAO with OCP = 58; PAO without OCP = 58). Compared to matched hips, unmatched hips were more likely to belong to male patients (unmatched = 27% male, matched = 12% male; *P* = .01). Unmatched hips also represented a wider distribution of alpha angles than matched hips (unmatched = 58.4 ± 18.2°, matched = 59.7 ± 11.1°; *P* = .02). For matched hips, the mean patient age at the time of surgery was 27.3 ± 5.5 years and was similar between treatment cohorts (*P* = .16). There were 102 (88%) females in the study: 51 (50%) in the PAO with OCP group, and 51 (50%) in the PAO without OCP group. Average follow-up was 4.1 ± 1.2 years.

Mean baseline patient characteristics and preoperative functional scores were similar between matched groups for all variables except HOOS symptoms, where the PAO with OCP group had a significantly lower preoperative score than the PAO without OCP group (53.0 ± 17.0 versus 60.8 ± 18.3, respectively; *P *= .001; Table 1). Mean preoperative mHHS was 60.3 (SD = 16.1) in the PAO with OCP group and 61.0 (SD = 17.7) in the PAO without OCP group (*P* = .53). Hip arthroscopy was performed in a total of 14 hips, 8 (14%) in the PAO with OCP cohort and 6 (10%) in the PAO without OCP cohort (Table 2).

**Table 1.** Baseline functional scores by procedure type (matched cohorts)

	Mean difference	PAO OCP (*N* = 58)	PAO only (*N* = 58)	*P*-value
HOOS pain	−4.6	55.1 ± 20.4	61.5 ± 20.0	.22
HOOS ADL	−6.7	65.3 ± 21.5	74.1 ± 20.2	.09
HOOS symptoms	−8.0	54.7 ± 18.6	64.5 ± 18.1	**.01**
HOOS sports and rec	−9.3	42.2 ± 24.2	52.0 ± 23.4	.09
HOOS QoL	−4.2	35.1 ± 20.7	40.4 ± 23.1	.33

Bold: statistically significant results

**Table 2.** Preoperative standardized differences of matched variables

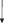	Standardized difference	PAO OCP (*N* = 58)	PAO only (*N* = 58)	*P*-value
Sex (female)	0	50 (81%)	50 (81%)	.99
LCEA	0.069	13.1 ± 7.4	12.6 ± 8.2	.75
Max Alpha	−0.010	59.7 ± 10.9	59.8 ± 11.4	.80
IRF	−0.014	38.1 ± 26.2	38.4 ± 21.5	.24
Hip scope	0.098	8 (14%)	6 (10%)	.78
mHHS	−0.039	60.3 ± 16.1	61.0 ± 17.7	.53

At final follow-up, there were no significant differences in the rates of clinical failure or reoperation between the two groups, and no significant differences in the outcome scores between groups. The rate of clinical failure in the PAO with OCP group was 22% (*n* = 13) compared to 14% (*n* = 8) in the PAO without OCP group (*P* = .23). There were two cases of revision arthroscopy, one case in each treatment group (revision rate = 1.7%). No hips were converted to THA. Both groups demonstrated improvement in final PROMs compared to baseline. There was no significant difference in final mHHS between the two cohorts (83.2 ± 16.2 PAO with OCP versus 84.1 ± 15.9 PAO without OCP, *P* = .74). Similarly, there were no significant differences in the final HOOS subscales for pain, activities of daily living, sports and recreation, or quality of life between the two cohorts (Table 3).

**Table 3.** Follow-up functional outcome scores by procedure type: 2- to 5-year follow-up

	*N* pairs	Paired diff	PAO OCP (*N* = 58)	PAO only (*N* = 58)	*P*-value
mHHS	58	−0.4	83.2 ± 16.2	84.1 ± 15.9	.74
Reoperation	58		1 (2%)	1 (2%)	.99
Clinical failure	58		13 (22%)	8 (14%)	.23
Composite outcome	58		13 (22%)	8 (14%)	.23
mHHS MCID	58		42 (72%)	46 (79%)	.37
mHHS PASS	58		37 (64%)	44 (76%)	.14
HOOS pain	56	−3.4	79.7 ± 20.1	83.4 ± 19.9	.51
HOOS ADL	57	−0.8	88.3 ± 16.8	88.8 ± 17.1	.81
HOOS symptoms	57	−4.1	72.9 ± 17.7	76.9 ± 20.3	.92
HOOS sports and rec	56	−2.7	72.9 ± 26.3	76.3 ± 22.8	.29
HOOS QoL	56	−5.1	63.8 ± 26.6	69.0 ± 24.0	.45

Values presented as mean ± SD, or *N* (%). Wilcoxon Signed-Rank test was used for all continuous outcomes, McNemar for reoperation and clinical failure.

## Discussion

To our knowledge, this is the largest and most rigorously matched study comparing outcomes of PAO with OCP and PAO without OCP. Using propensity score matching, we identified and compared patients with similar baseline characteristics affecting the likelihood of undergoing OCP and found no significant differences in PROMs (mHHS and HOOS subscores), reoperation rates, or clinical failure between the groups at short-term follow-up. Our findings suggest that PAO with OCP is not superior to PAO alone and may not be routinely required for all patients with acetabular dysplasia at short-term follow-up.

Consistent with these findings, a 2016 study by Goronzy *et al*. [9] investigated outcomes of combined PAO and OCP versus PAO without OCP in 85 hips with a mean 5.2 years follow-up and found no significant differences in postoperative WOMAC scores or rates of conversion to THA between treatment groups. In that study, the preoperative alpha angle was elevated in the combined treatment group (49° versus 63°; *P* = .009), but there was no difference in postoperative alpha angles between the two groups (39° in isolated PAO versus 37° in PAO/OCP; *P* = .134). This study was retrospective and did not match or control for baseline characteristics between groups.

While our short-term follow-up study suggests no significant difference between either approach, other studies have concluded that a combined approach may offer superior results in the short term. A study [20] of 48 hips followed for a mean of 3.2 years compared the outcomes of isolated PAO, PAO with arthroscopy, and PAO with arthrotomy. A subanalysis compared outcomes by the degree of intraarticular intervention: OCP and labral repair were considered major interventions, labral debridement was considered a minor intervention. There were 27 patients who underwent major intraarticular intervention. The major-, minor-, and no-intervention groups did not differ significantly by age, sex, BMI, prior surgery or IRF, but the major intervention group had significantly greater baseline alpha angles (*P* < .001). For each physical outcome measure (UCLA, mHHS, WOMAC, HOOS) the major intervention group demonstrated the greatest improvements compared to baseline (*P* = .007). There were no differences in reoperation-free survival between any of the groups. The authors concluded that major intraarticular intervention (OCP or labral repair) at the time of PAO results in superior improvement in postoperative measures of pain and function at short-term follow up. The authors recommended that intraarticular pathology should be treated at the time of PAO to improve outcomes. However, since there were higher alpha angles in the major intervention group, it is unclear if selection bias accounts for the significantly greater improvement in PROMs compared to baseline. There was also no distinction within the major intervention group between patients undergoing labral repair and OCP, or whether these intra-articular interventions were performed through arthroscopy or arthrotomy. In addition to patients with acetabular dysplasia, the study included patients undergoing PAO for FAI. The study was also limited in its subgroup analyses due to a relatively small sample size. Our study was able to match preoperative alpha angles and other clinically important baseline characteristics (Table 1) while analyzing a much larger cohort (*n* = 116) of patients undergoing PAO than had previously been evaluated.

It is possible that the full benefits of PAO/OCP are not appreciated in the short term and that the combined approach is superior for patients in the long term. Wells *et al*. [17] studied 154 hips followed for a mean of 10.3 years after PAO and found that the combined PAO/OCP was associated with a decreased risk of failure (defined as THA, a WOMAC pain score of ≥10, and/or an mHHS of ≤70) compared to PAO alone. The study included 66 (42%) hips undergoing the combined procedure. There were 32 failures at final follow-up, 7 of which (22%) occurred in the setting of a combined OCP. The failure rate in the combined procedure group was 10.6% compared with 28.4% in the group that did not undergo OCP. Undergoing PAO with OCP was associated with a 73% reduction in the odds (OR: 0.27; *P* = .016) of failure at final follow-up compared with hips that did not undergo OCP. The authors concluded that not undergoing concurrent OCP was predictive of a failed outcome. Although we did not find a significant difference in outcomes between our two cohorts at 2- to 5-year follow-up, there may also be little downside to the combined approach if its long-term effect could be valuable.

In response to the mixed literature on outcomes for PAO with OCP, surgeons have performed OCP selectively based on different criteria (i.e. preoperative ROM, intraoperative ROM, and/or femoral head-neck junction morphology, etc.) and surgeon preference. The present study provides—through propensity matching—the best-controlled results to date on the question of short-term outcomes of the combined surgical approach.

This study has several limitations. While propensity matching is a powerful statistical method, a randomized trial would be ideal in determining the superiority or noninferiority of different surgical approaches to the dysplastic hip. Propensity score matching reduces the bias associated with comparison between non-randomized groups by controlling for known differences between cohorts. By using strict match criteria, our study was able to match hips with similar preoperative likelihoods of undergoing OCP but we were unable to match over 50% of hips that were initially included. The <50% match rate for the cohort indicates that our results are best applied in patients for whom the surgeon feels the indication for PAO with or without OCP is equivocal, as these patients will have similar baseline characteristics. Our findings are therefore not generalizable to patients at the extremes of ROM or more significant degrees of proximal femoral asphericity.

An additional weakness of propensity matching is the inability to analyze subgroups based on individual characteristics (e.g. maximum alpha angle >65°), and there is no way to compare outcomes in patients with and without hip arthroscopy. This is because the propensity score is derived from several patient parameters, rather than simple matching. Paired hips are not matched for each parameter and are thus precluded from subcategorization. Further work is necessary to determine if certain patient subpopulations would still benefit from an OCP at the time of PAO. This study examines a cohort with short-term follow-up (mean follow-up = 4.1 years). FAI has been reported to develop up to several years after PAO, and an aspherical femoral head is associated with increased risk of PAO failure at 10 year follow-up [21]. CAM lesions are unlikely to develop de novo post-PAO; rather, patients may present with FAI symptoms later due to pre-existing asphericity or overcorrection during the procedure. It is possible that superiority of the combined approach is only appreciated after extended follow-up due to a slight improvement in survivorship afforded by the OCP. A similar study with greater follow-up and the option for subgroup analysis will be valuable in comparing the isolated and combined surgical approaches over the long-term. Additionally, our study prospectively collected data that unfortunately did not include information on femoral or acetabular version which could affect indications for OCP. Finally, this multicenter cohort includes several expert hip preservation surgeons, all of whom are very experienced in performing PAO for typical acetabular dysplasia. This is important in considering the generalizability of these results.

## Conclusions

In this propensity-matched study comparing PAO/OCP with PAO alone, our data suggest noninferiority of either approach in addressing symptomatic acetabular dysplasia. Additional studies should seek to identify individual patient characteristics that predispose patients to a favorable outcome with the combined surgical approach.

## Data Availability

The data underlying this article cannot be publicly shared because patient consent was obtained for enrollment and usage within the study group. Aggregate data can be shared upon reasonable request to the corresponding author.
